# Efavirenz induces DNA damage response pathway in lung cancer

**DOI:** 10.18632/oncotarget.27725

**Published:** 2020-10-13

**Authors:** Rahaba Marima, Rodney Hull, Zodwa Dlamini, Clement Penny

**Affiliations:** ^1^SA-MRC/UP Precision Prevention and Novel Drug Targets for HIV-Associated Cancers Extramural Unit, Pan African Cancer Research Institute, Faculty of Health Sciences, University of Pretoria, Hatfield 0028, South Africa; ^2^Department of Internal Medicine, School of Clinical Medicine, Faculty of Health Sciences, University of the Witwatersrand, Parktown, 2193, South Africa

**Keywords:** efavirenz, cell cycle, differential gene expression, DNA damage response pathway, lung cancer

## Abstract

The cell-cycle related genes are potential gene targets in understanding the effects of efavirenz (EFV) in lung cancer. The present study aimed at investigating the expression changes of cell-cycle related genes in response to EFV drug treatment in human non-small cell lung carcinoma (A549) and normal lung fibroblast (MRC-5) cells. The loss in nuclear integrity in response to EFV was detected by 4′, 6-diamidino-2-phenylindole (DAPI) staining. Gene expression profiling was performed using human cell cycle PathwayFinder RT^2^ Profiler™ PCR Array. The expression changes of 84 genes key to the cell cycle pathway in humans following EFV treatment was examined. The R^2^ PCR Array analysis revealed a change in expression of selected gene targets (including MAD2L2, CASP3, AURKB). This change in gene expression was at least a two-fold between test (EFV treated) and the control. RT-qPCR confirmed the PCR array data. In addition to this, the ATM signaling pathway was shown to be upregulated following EFV treatment in MRC-5 cells. In particular, ATM’s upstream activation resulted in p53 upregulation in normal lung fibroblasts. Interestingly, the p53 signaling pathway was activated irrespective of the repressed ATM pathway in A549 cells as revealed by the Ingenuity Pathway Analysis (IPA). These EFV effects are similar to those of ionizing radiation and this suggests that EFV has anti-tumour properties.

## INTRODUCTION

The non-nucleoside reverse transcriptase inhibitor (NNRTI) efavirenz (EFV) is frequently used in human immunodeficiency virus (HIV) treatment, and forms part of the first-line Highly Active Antiretroviral Treatment (HAART) treatment against HIV/AIDS [1]. However, EFV has selective cytotoxic effects against different cancer cells [2, 3]. This includes cancers such as colorectal, glioblastoma, and pancreatic, while sparing human primary fibroblast cells [2]. Tumour growth in mouse models treated with EFV was also shown to be reduced [4, 5]. Jin et al., (2016) also revealed that EFV reduced proliferation of neuronal stem cell and increased apoptosis by increasing the expression of BAX and CASP3 [6]. Moraes-Filho et al., (2017) also demonstrated that EFV at high doses induced genotoxicity in Drosophila Melanongaster [7].

EFV also causes morphological changes in cells [8]. Xulu and Hosie (2017) showed that ARV drugs including EFV caused apoptosis in the Human Squamous Cell carcinoma from Uterine Cervix (HCS-2) cells and observed a change in morphological features such as rounding-up of cells, retraction of filopodia, blebbing and maintenance of plasma membrane integrity- characteristic features of apoptosis [9, 10]. In addition to EFV, the potential use of HAART components as anti-cancer agents is an evolving subject. For example, the HIV protease inhibitor nelfinavir was shown to be highly active on a variety of human cancer cells [11–15], and has been tested in several clinical studies on cancer patients [11, 16–18]. A recent clinical trial study also indicated that the addition of nelfinavir as a putative radiosensitizer with concurrent chemoradiotherapy in patients with locally advanced non–small cell lung cancer (NSCLC) may improve clinical efficacy and outcomes [19].

On the other hand, the molecular basis of lung cancer is heterogeneous and complex. Understanding the genetic and molecular alterations and their functional significance is rapidly influencing the potential impact molecular markers have on lung cancer diagnosis, prognosis and treatment [20]. Furthermore, the numbers of HIV-positive patients with lung cancer as a leading non-AIDS defining cancer (NADC) will most likely increase over the next two decades [21, 22]. This highlights the importance of further research on lung cancer, and the HIV epidemic as well as the potential interactions between the two diseases as the number of individuals with both diseases increases [21].

The South African (SA) healthcare system has to deal with the largest HIV burden in the world, resulting in the largest HAART programme globally, and a high prevalence of NADCs in the HAART era [23]. Additionally, lung adenocarcinoma has been shown to be the most common form of NADCs [21]. To date, the relationship between the use of HAART and lung carcinogenesis is poorly understood. While the deregulation of the cell-cycle is one of the hallmarks of lung cancer, including lung cancer [24]. Mitotic cell cycle progression is accomplished through a series of events, DNA replication (S phase) and mitosis (M phase) separated by gap phases G1 and G2 [25]. Cyclin/Cyclin-dependent kinases (CDKs) are key regulatory elements of the cells’ progression through the cell cycle. Precise activation and inactivation of CDKs at specific points in the cell cycle are required for orderly cell division [26]. Cyclin-CDK inhibitors (CKIs), such as p16Ink4a, p15Ink4b, p27Kip1, and p21Cip1, are involved in the negative regulation of CDK activities, thus negatively regulating the cell cycle [26, 27]. Because the cell cycle is a tightly regulated process, eukaryotic cells respond to external stimuli such as DNA damage by activating signaling pathways that promote cell cycle arrest and DNA repair [28]. In response to DNA damage, the checkpoint kinase ATM phosphorylates and activates Chk2, which in turn directly phosphorylates and activates TP53 tumour suppressor protein. TP53 and its transcriptional targets play an important role in both G1 and G2/M checkpoints.

A previous study performed by our group, involved assessing the effects of EFV on lung cancer cells at the cellular level on the physiological health of treated cells. This study aimed at elucidating the effects EFV has on lung cancer in *in vitro* cell-line models. Findings revealed that EFV induced S-phase cell-cycle arrest and had anti-proliferative effects. To date, several studies including Hecht et al., (2018) have revealed the cytotoxic effects of EFV against several cancer cells [3], but to our knowledge, no study yet has shown the anti-proliferative effects of EFV on lung epithelial cancer cells in relation to primary lung fibroblast cells. In conjunction with preceding studies on EFV’s cyto-and-genotoxicity, this study is the first to reveal EFV mediated ATM/ATR genotoxicity in lung cells.

## RESULTS

### Evaluation of nuclear morphology pre- and post EFV treatment, using DAPI staining

**Figure 1:**
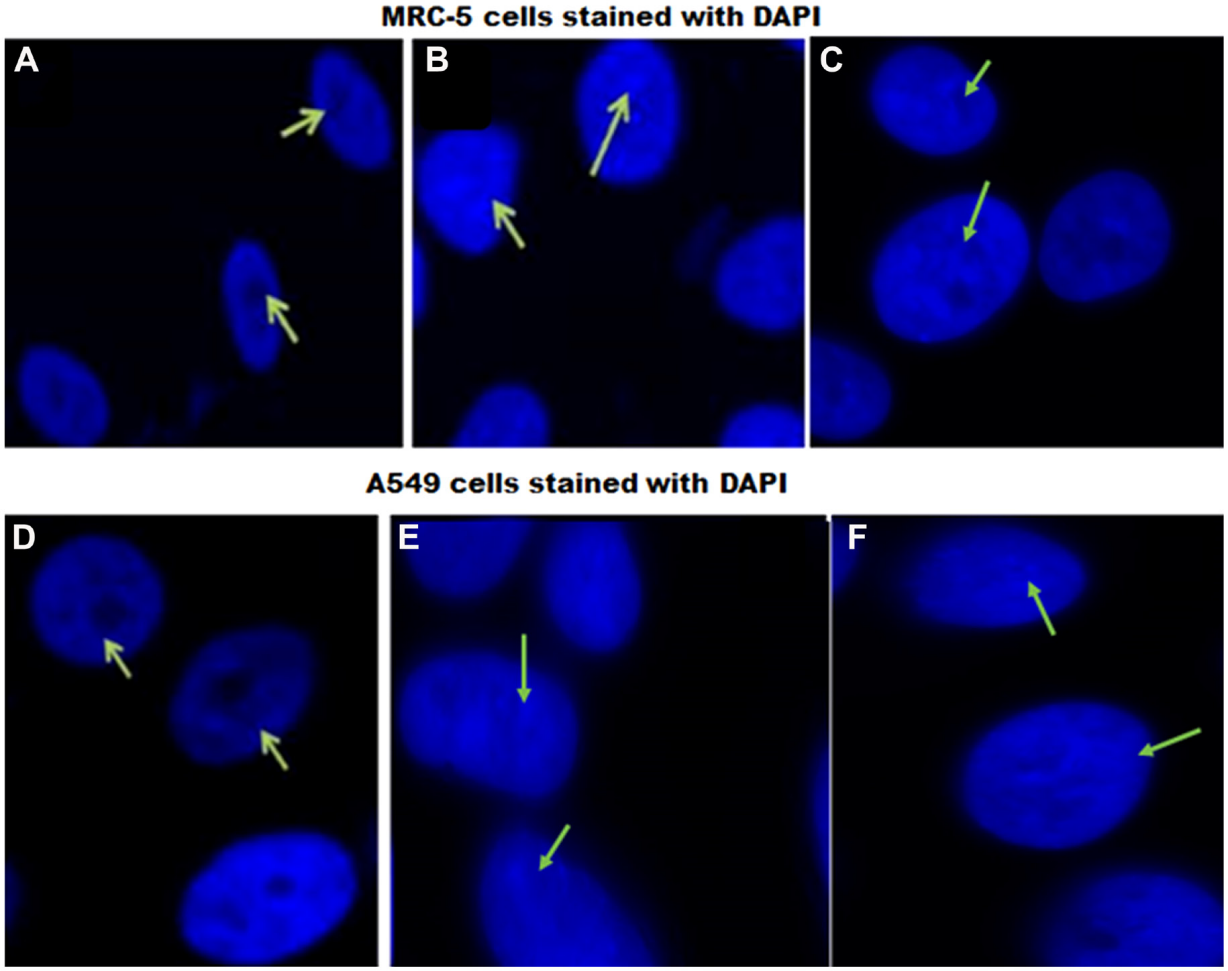
DAPI staining of MRC-5 and A549 cells in response to EFV. Changes in morphology were assessed in ARV drug treated relative to control cells. (**A**) and (**D**) represent control cells, (**B**) and (**E**) show 13 μM EFV treated cells, while (**C**) and (**F**) illustrate 50 μM EFV treated cells. Green arrows point to changes in the nucleus such as DNA fragmentation and chromatin condensation in EFV drug treated (B and D, and C and F) relative to vehicle control cells (A and D) (Original Magnification, 63×).

DAPI staining was used to determine morphological and nuclear changes such as DNA fragmentation and chromatin condensation in MRC-5 and A549 cells in response to EFV. This analysis is presented below, Figure 1. Staining indicated that DNA fragmentation and chromatin condensation occurs in cells treated with EFV. Control (vehicle) treated cells did not show signs of DNA fragmentation or chromatin condensation.

### Profiling of the human cell cycle gene response post EFV treatment in human non-small cell lung carcinoma (NSCLC) cells

#### Human cell-cycle PCR arrays

Following on the aforementioned observations relating to loss of nuclear integrity, a specific gene panel was employed here to more specifically interrogate changes in the expression of cell cycle related genes in response to EFV treatment. These findings are presented below.

#### Assessment of quality control (QDC)

**Figure 2:**
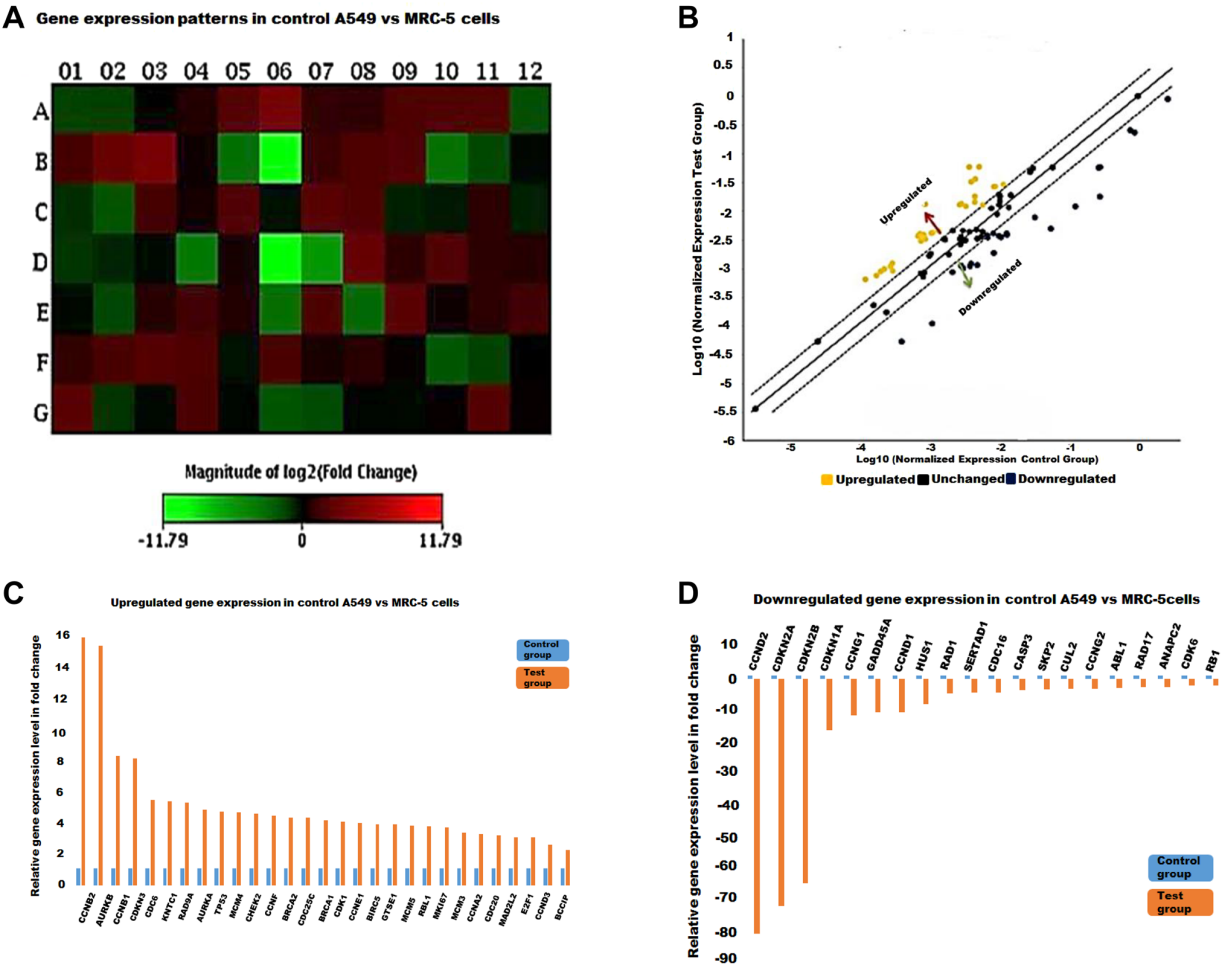
The cell cycle gene expression profile in control A549 vs MRC-5 cells. (**A**) A heat map showing red and green blocks representing increased or decreased gene expression in the test group against the control group respectively. (**B**) The yellow and blue dots represent up-regulated and down-regulated genes as illustrated in the scatter plot. Genes that increased (**C**) and decreased (**D**) with at least a two-fold differential expression in test MRC-5 against the control A549 cells represented by histograms.

**Figure 3:**
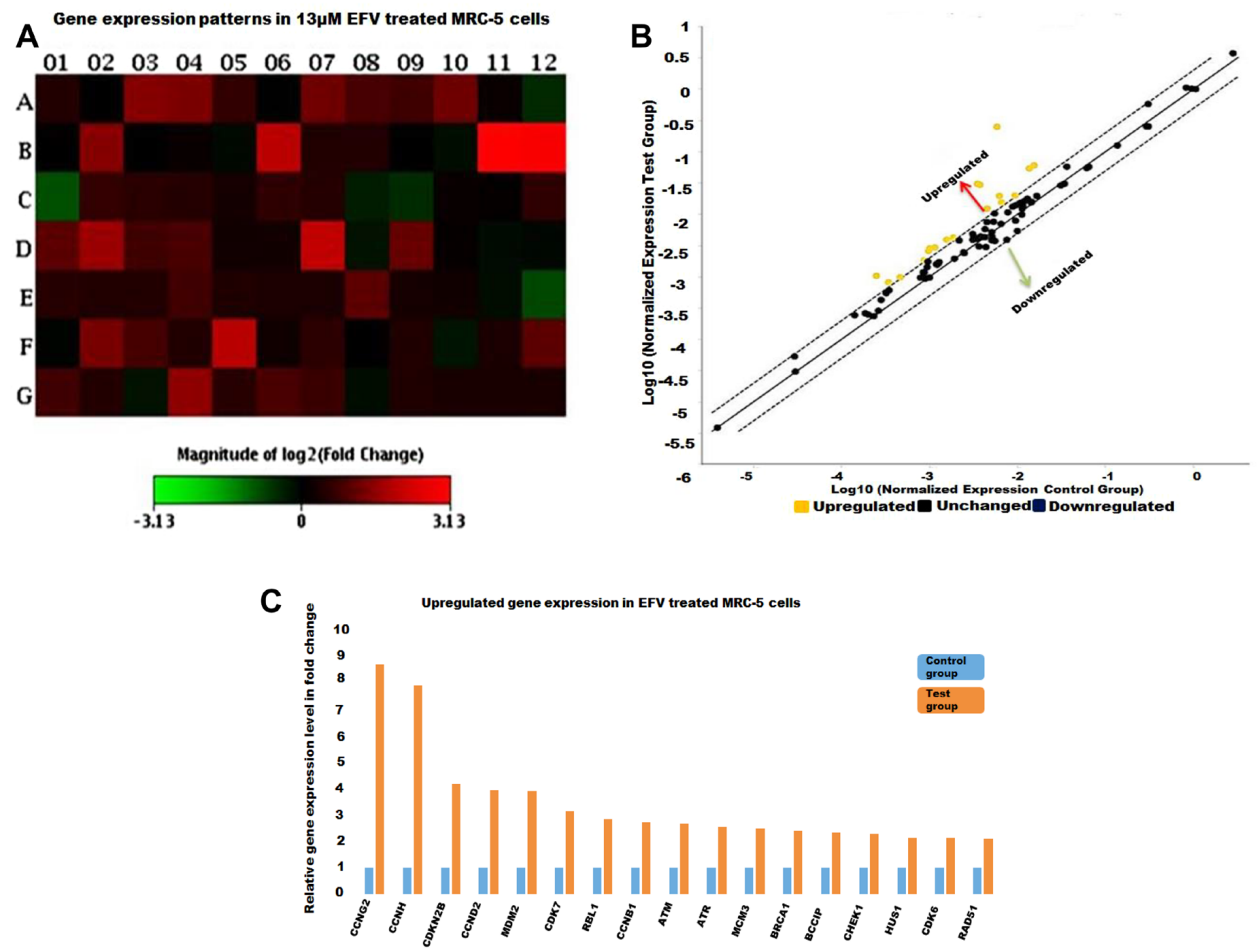
The cell cycle gene expression profile EFV treated MRC-5 cells. (**A**) A heat map with the red and green blocks representing increased or decreased gene expression in the test group against the control group respectively. (**B**) The yellow dots stands for up-regulated genes as illustrated in the scatter plot. No significantly downregulated genes are depicted in this scatter plot. (**C**) Genes that increased in expression by at least two-fold in test (EFV treated) against the control group represented as histograms.

**Figure 4:**
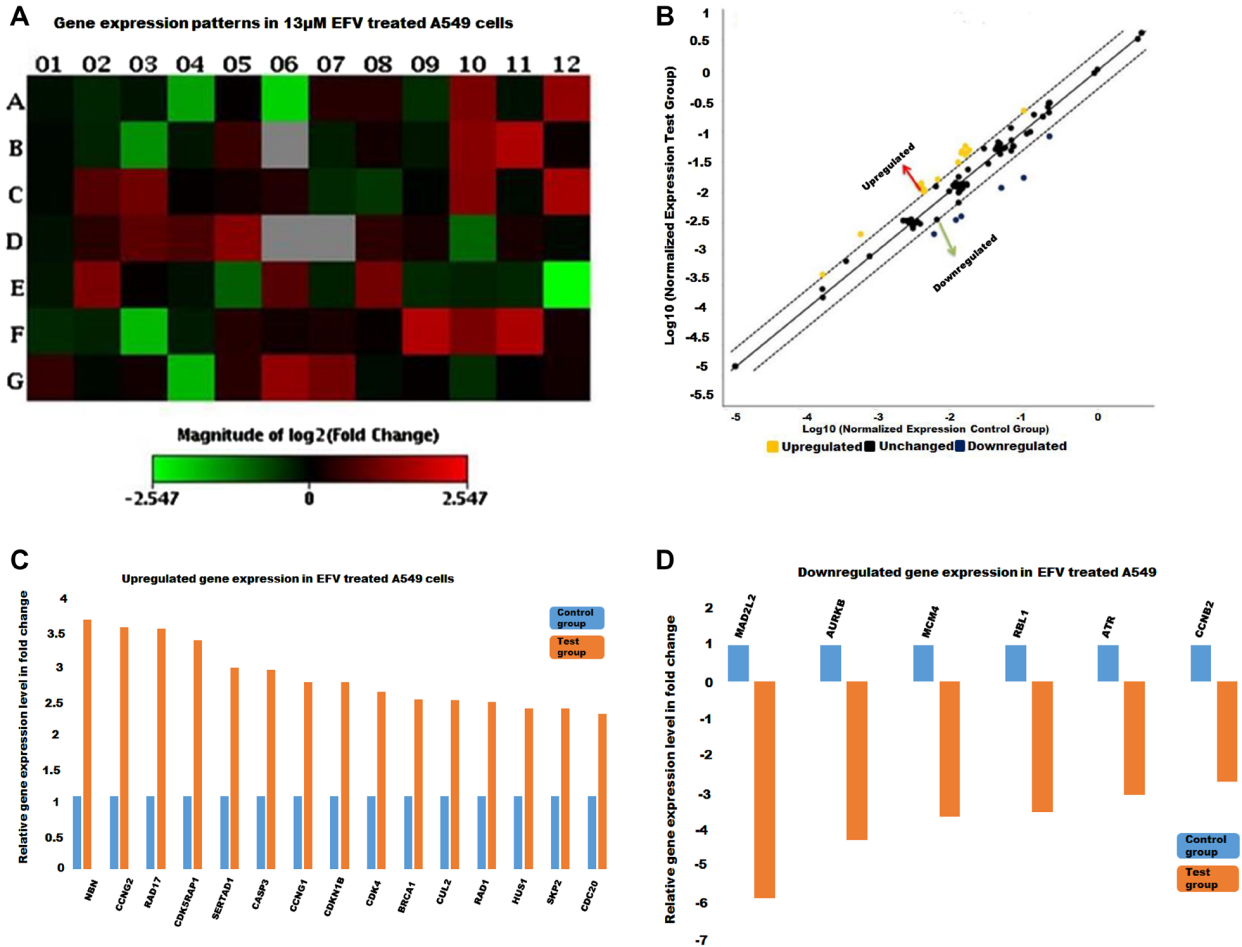
The cell cycle gene expression profile in EFV treated A549 cells. (**A**) A heat map with the red and green blocks representing increased or decreased gene expression in test group against control group respectively. (**B**) The yellow and blue dots stand for up-regulated and down-regulated genes as illustrated in the scatter plot. Genes whose expression increased (**C**) and decreased (**D**) with at least a two-fold in test (EFV treated) against the control group represented by the histograms.

PCR arrays, incorporating 84 genes related to the cell cycle were profiled on 4 samples, including methanol control and 13 μM EFV treatment on both MRC-5 cells and A549 cells. The results of the gene expression arrays were analysed by the GeneGlobe program (Qiagen), by comparing the normalised fold changes of the test group against the control group. All four PCR Arrays were subjected to data quality control checks (using the GeneGlobe Program, for monitoring genomic DNA contamination (GDC), the first strand synthesis (RTC) as well as real-time PCR efficiency (PPC). All four arrays passed these checks, Table 1. Figures 2–4 represent gene expression profiles in test (EFV treated) vs control groups.

**Table 1:** RT^2^ PCR array quality check by proprietary controls

Quality test performed	Test result
1. PCR Array Reproducibility	All Samples Passed
2. RT Efficiency	All Samples Passed
3. Genomic DNA Contamination	None detected-Passed

### EFV treatment modulates the expression of genes related to the cell cycle in lung cancer cells (A549) and in normal lung cells (MRC-5) groups

In the present study, gene expression in the control tumour vs the normal lung cells’ array, p53 was 4.32 fold up-regulated. However, most of the CDKIs (which also act as down-stream effectors of p53), were significantly down regulated and these included CDKN3, p21, p15 and most significantly p16. CASP3, an effector caspase in apoptosis was also significantly down regulated. In addition, GADD45A, which is also triggered by p53 in response to DNA damage and growth-arrest was significantly down regulated (-10.7 fold). Cyclin G1 and G2, which are both induced following DNA damage and maintain the p53 dependent cell cycle arrest and RAD DNA repair genes were also down-regulated. On the other hand, the cyclins such as cyclin A, B, D3, E and F and CDK1 were found to be significantly upregulated. Also, survivin (BIRC5), a pro-survival gene was up-regulated here. Furthermore, the E2F1 transcription factor, important for the transcription of S-phase genes was up-regulated. Additionally, genes required for S-phase and DNA replication, the MCM gene family was significantly up-regulated. Furthermore, the AURK family (A and B), as well as MAD2L2 (involved in the mitotic spindle-checkpoint), were significantly up-regulated.

Prior to treatment a significant number of genes are shown to be dysregulated, either up-or down, and were represented as shifting from the median-solid line, in the scatter plots as shown in Figures 2–4. Referring to Figure 2, the upregulated genes included cyclin/CDK complexes, while the down-regulated genes included growth-arrest genes, such as p21 and GADD45A. On the other hand, RT-PCR arrays indicated a general up-regulation in the transcription of cell cycle genes in MRC-5 cells treated with EFV while most genes remained in a normal (unchanged) state, Figure 3. EFV treatment of A549 cells led to the increase in the transcription of some cell cycle genes and a decrease in the transcription of others, Figure 4. However, the expression of most genes were observed to remain the same when normalized expression values for treated cells were compared to the vehicle control cancerous cells.

### Validation of selected cell-cycle associated gene targets using real-time quantitative polymerase chain reaction (RT-qPCR)

The Real-Time quantitative Polymerase Chain Reaction (RT-qPCR), a highly sensitive method for gene expression studies was used here to assess and confirm the relative gene expression levels of selected target genes from the cell-cycle arrays.

A number of candidate genes were shown to be differentially expressed, Figures 2–4. Based on their differential expression across test and control groups and their role in cell proliferation, three differentially expressed genes were selected, shown to be either up-or-down-regulated, from the gene array studies. The three genes selected were Mitotic Arrest Deficient-Like 2 (MAD2L2) which functions at the cell-cycle checkpoint, Caspase 3 (CASP3) which is apoptosis related and Aurora Kinase B (AURKB), a mitotic gene.

Following fold change calculations (using GenGlobe), the resulting data was then exported to GraphPad Prism 5, for further statistical analysis, plotting test against control, for all three selected target genes. Results are represented as fold changes in histograms.

### Analysis of MAD2L2, CASP3 and AURKB gene expression levels before and after ARV treatment

**Figure 5:**
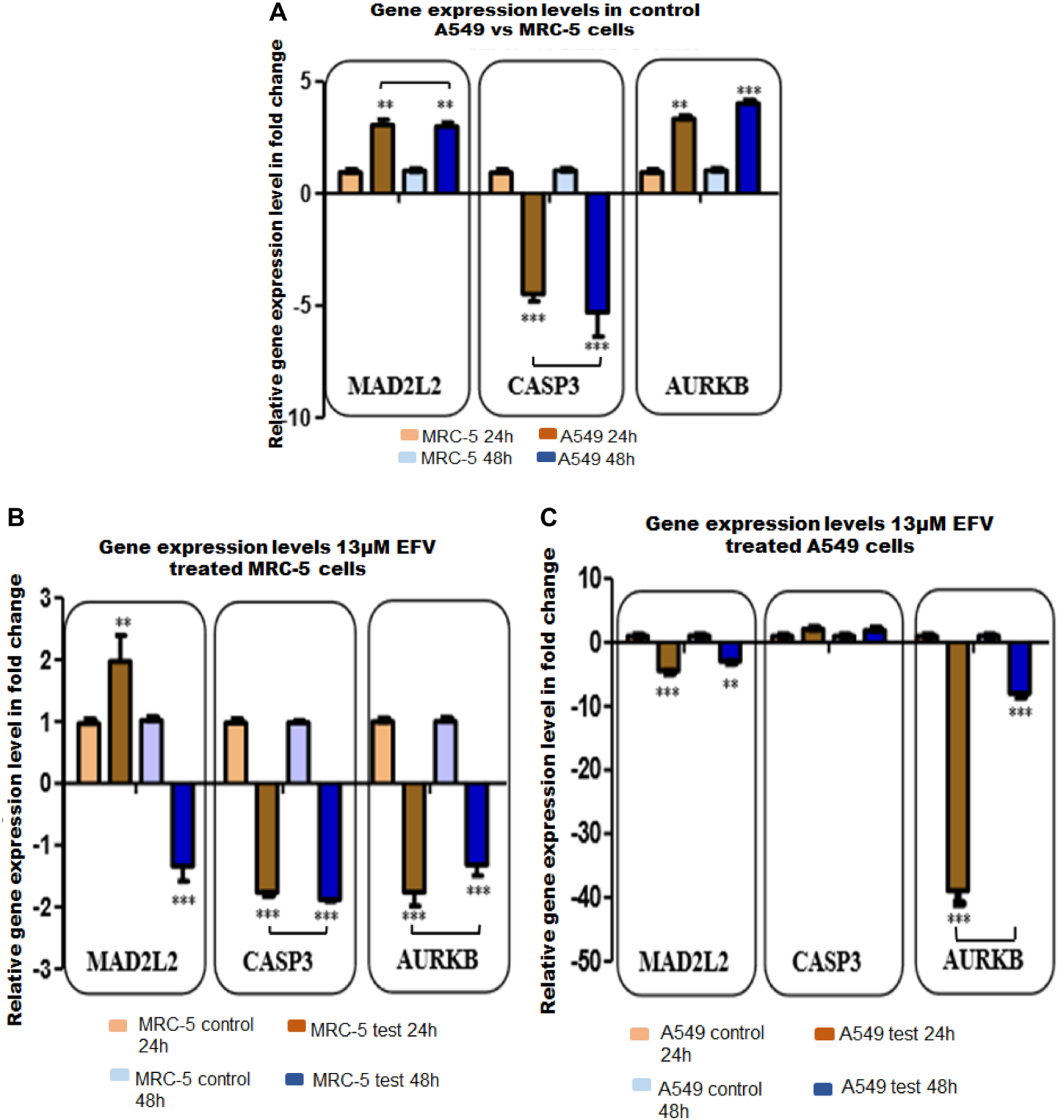
The validation for selected target genes by qPCR (**A**–**C**) in test (13 μM EFV treated) and control cells at 24 h and 48 h. (A) Represents target (MAD2L2, CASP3, AURKB) gene expression (GE) changes expressed as fold change in control A549 vs MRC-5 cells. (B) Shows target GE in fold change in EFV treated MRC-5 cells. (C) Illustrate target GE in fold change in EFV treated A549 cells. All experiments were performed in triplicate at least three times. ^*^ *p* < 0.05, ^**^ *p* < 0.01, *p* < 0.001.

Prior to assessing the effects of ARVs on target gene expression, the expression levels of MAD2L2, CASP3 and AURKB were first assessed in control A549 vs MRC-5 cells, respectively. Both MAD2L2 (~3 fold) and AURKB (3–4 fold) at 24 h and 48 h were significantly upregulated in A549 cells relative to the normal MRC-5 fibroblasts. Caspase 3 in contrast was significantly down-regulated (~ -5 fold) at both 24 h and 48 h in A549 lung cancer cells, Figure 5A. In EFV treated MRC-5 cells, MAD2L2 was significantly upregulated (~2 fold) at 24 h, followed by a –1.34 down-regulation at 48 h. In contrast, CASP3 (~ –1.8 fold) and AURKB (~ –1.5 fold) were down-regulated at 24 h and 48 h, as shown in Figure 5B. In EFV treated A549 lung cancer cells, EFV significantly decreased expression levels of both MAD2L2 (fold changes, ^**^ *p* < 0.01 and ^***^ *p* < 0.001) and AURKB (fold changes, *p* < 0.001) genes at 24 h and 48 h. The expression of CASP3, though upregulated (~2 fold) at 24 h and 48 h post treatment, was not statistically significant, Figure 5C.

### Ingenuity pathway analysis (IPA)

**Figure 6:**
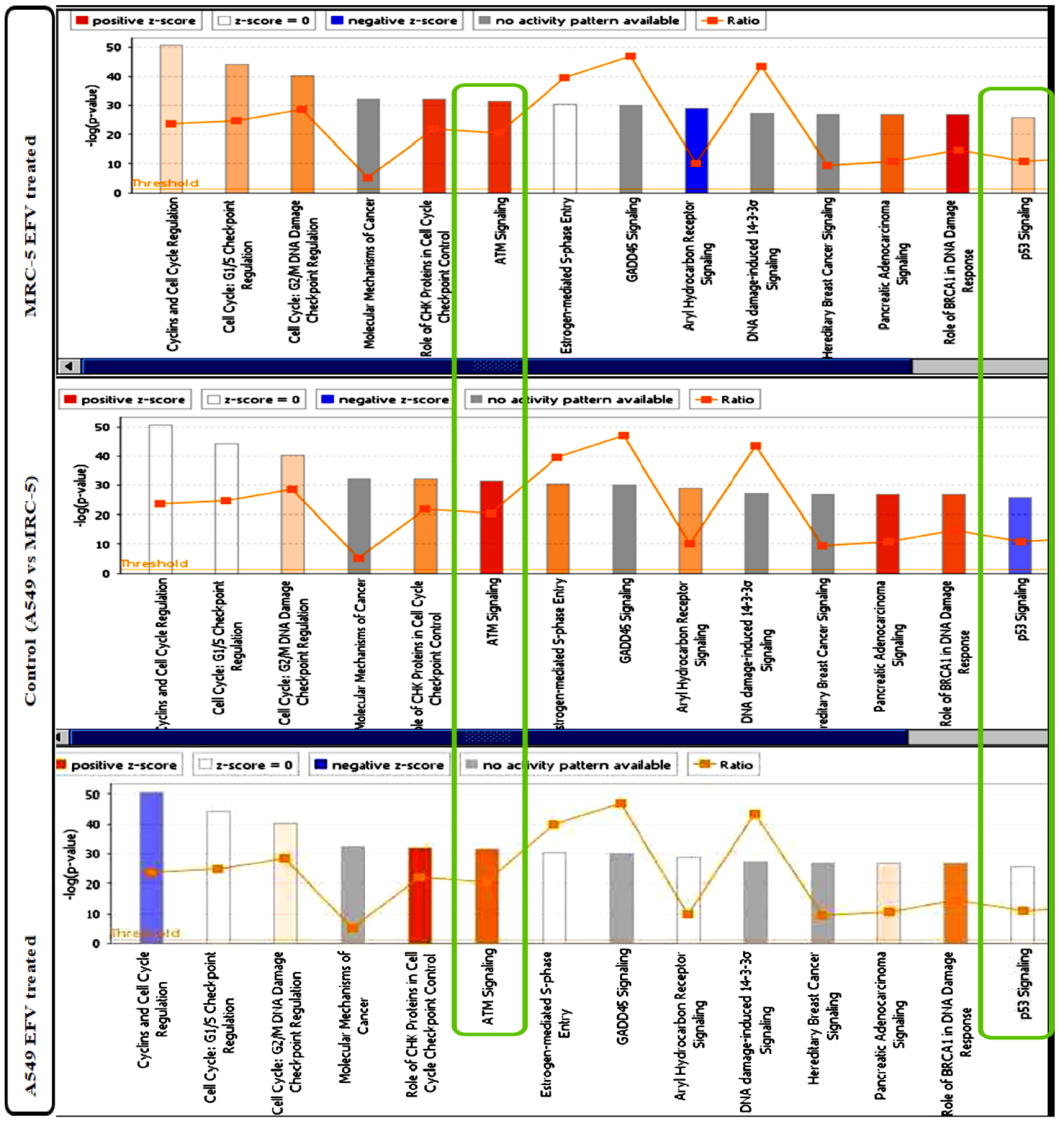
IPA Canonical Pathway analysis of differentially expressed genes in EFV treated vs control cells. The orange bars indicate activated pathways, while blue bars indicate repressed pathways. The colour intensity is proportional to the degree of in/activation. The green boxes highlight pathways of interest, the ATM signaling pathway and the p53 signaling pathway. Being upstream of the p53 pathway, the ATM signaling pathway does not activate its down-stream effector p53 pathway in control cells, compared to EFV-treated cells.

**Figure 7:**
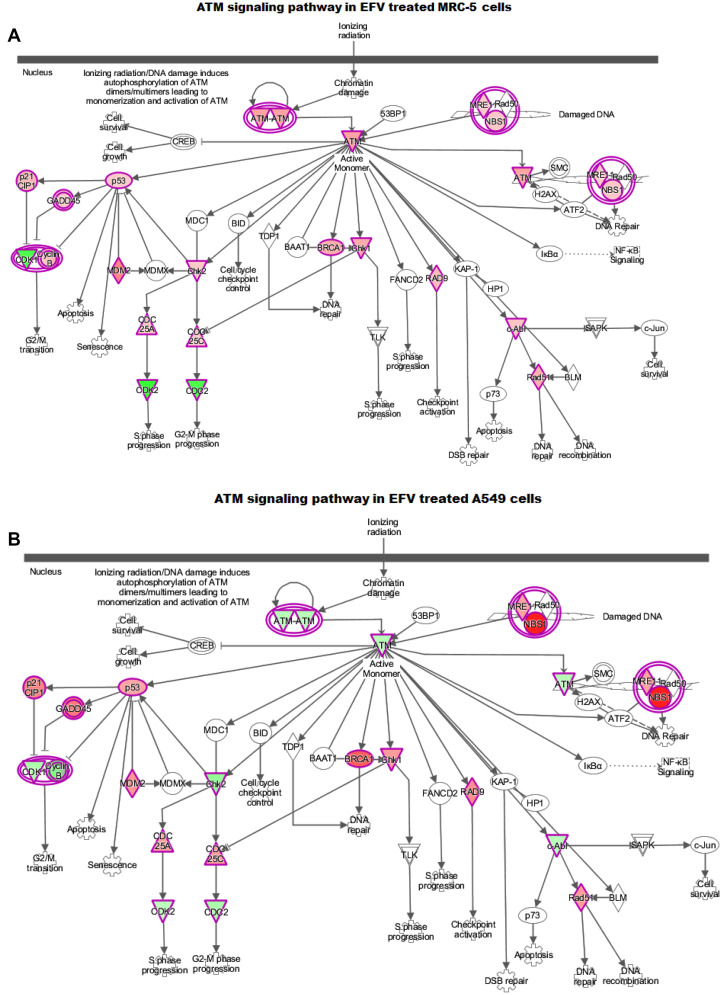
IPA ATM-signaling pathway in (**A**) EFV treated MRC-5 and (**B**) A549 cells. The green and the red colours indicate down and up-regulation. The activated p53 irrespective of the expression levels of ATM activates its downstream targets such as p21, GADD45A, BRCA1 and NBS, inhibiting cell cycle progression and initiating DNA repair and growth arrest mechanisms.

Qiagens’s Ingenuity Pathway Analysis (IPA) has been widely used to model, analyse and understand complex biological systems. In this study, the canonical pathways and core analysis functions of IPA were used to help build a more complete regulatory picture to better elucidate the biology underlying the studied gene expression profiles. The analyses are represented as bar graphs, with the z-score referring to the activation state, either up- (orange bars) or down (blue bars) regulation of the pathway, in the canonical pathways, Figure 6. Green and red colours in the core analysis represent the down and up regulated genes respectively, Figure 7. The ATM (Figure 7A) and the p53 (Figure 7A and 7B) signalling pathways are key pathways in DNA damage response (DDR), and have been shown to be activated following EFV treatment.

## DISCUSSION

The loss of nuclear integrity in response to EFV in both MRC-5 and A549 cells is observed. This suggests EFV’s potential damage to the genomic DNA. Progression through the cell-cycle is a finely regulated process, wherein cyclins and CDKs promote cell-cycle progression, whilst the CDKIs inhibit progression. The balance between cyclins/CDKs and CDKIs is essential in maintaining cellular homeostasis, and determines cell fate, that is, proliferation, senescence or cell death (apoptosis). To ensure integrity of DNA replication and cell division, cell-cycle checkpoints exist at the key transition points of G1/S and G2/M, respectively. As the CDKIs act at multiple phases of the cycle, they are particularly important at these checkpoints. Prior to the synthesis of DNA (during the S-phase), the G1/S checkpoint allows for the monitoring of DNA integrity before the cell’s DNA is replicated. The G2/M checkpoint allows the cell-cycle to pause prior to mitotic cell division. P53 is an important regulator of these checkpoints [29].

The treatment of MRC-5 and A549 cells with EFV alters the gene expression of important factors that are essential in the maintenance of genomic stability in relation to the cell cycle. This is particularly observed in the cancerous cells, with the significant down-regulation of AURKB and MAD2L2. Even though the normal p53 (1.02 fold) expression was shown here, p27, CASP3, Cyclin G1 and G2, NBN, RAD1 and RAD17 were significantly up-regulated. The E2F4 transcription factor, important for the transcription of S-phase genes, was also ~2 fold up-regulated. Interestingly, the S-phase and DNA replication genes were downregulated; MCM4 in particular was –3.65 significantly down-regulated. These EFV-treated genotypic alterations observed here are characteristic of anti-tumour properties. Referring to the IPA analysis, EFV exhibits radiosensitizing effects. Depending on the severity of these effects in the physiological health of normal cells, EFV poses as a promising drug that can be used in synergy with chemo/radiotherapy. Additionally, EFV has an excellent safety profile compared to classical chemotherapy against cancer [30]. Future investigation would involve establishing the level of double strand breaks following EFV treatment and can be quantified using Immunofluorescence and Western blot analysis using an antibody targeted against γH2A-X, a typical DSBs marker. Comet assays would allow the evaluation of DNA damage associated with alkylation, intercalation, and oxidation [31]. Posttranscriptional gene regulation targeted by EFV in lung cells would also be interesting to pursue.

## MATERIALS AND METHODS

### Cell culture

The lung cell lines MRC-5 and A549 were purchased from the American Type Culture Collection (ATCC). The MRC-5 and A549 cells were maintained in Dulbecco’s modified Eagle’s medium (DMEM) (Sigma-Aldrich; Merck KGaA, Darmstadt, Germany), media supplemented with 10% (v/v) fetal bovine serum (FBS) (Gibco; Thermo Fisher Scientific, Inc., Waltham, MA, USA) and 1% (v/v) penicillin/streptomycin at 37°C in a humidified atmosphere containing 5% CO_2_. For cellular health and nuclear integrity analysis, lung cells were treated with 13 μM and 50 μM EFV. For cell cycle PathwayFinder RT^2^ PCR Array analysis, the MRC-5 and A549 cells growing at an exponential phase were treated with 13 μM EFV for 48 h, subsequent to cell cycle synchronisation. For validation study, the control groups were also included. These two concentrations were selected as they represent a clinical plasma level dose [32] and an experimental dose, as described in Marima et al., (2020) submitted.

### DNA staining using 4, 6-diamidino-2-phenylindole (DAPI)

Cells were first fixed by 4% paraformaldehyde (Aladdin, Shanghai, China) in the microfluidic channels at room temperature for 10 min, washed with phosphate buffered saline (PBS) three times, permeabilized by 0.3% Triton X-100 (Aladdin) for 10 min, washed with PBS again three times, and finally stained by 4′,6-diamidino-2-phenylindole (DAPI) (Meilune Biotech) for 15 min. The cells were viewed on the Zeiss LSM 780 confocal microscope.

### Human cell-cycle gene (PCR) arrays

A human cell cycle PathwayFinder™ RT^2^ Profiler™ PCR Array (PAHS-020Z, Qiagen, Frederick, MD., USA) was used to screen a panel of 84 genes representative of the human cell cycle pathway in human lung cells (Table 2). Total RNA was isolated from 13 μM EFV treated cells and 0.1% (v/v) vehicle control cells using Qiagen RNeasy Mini Kit by following manufacturer’s protocol. RNA was quantified using a Nanodrop (Nanodrop Technologies) and the quality was assessed by visualizing 18 S and 28 S ribosomal RNA bands separated through 1% agarose with ethidium bromide staining. The first-strand cDNA was mixed with 2 × RT^2^ SYBR Green qPCR Master Mix and ddH2O. The qPCR was performed on an Applied Biosystems (ABI) 7500 according to the RT^2^ Profiler PCR Array instructions under the following conditions: 95°C for 10 min, then 40 cycles at 95°C for 15 sec and 60°C for 1 min. Aside from the pathway-focused genes, the panel contains 5 housekeeping genes (Table 2, H1 to 5) that were used for normalization of the sample data and additionally a panel of proprietary controls that monitor genomic DNA contamination (GDC: Table 2, H6), first strand synthesis (RTC: Table 2, H7 to 9) and real-time PCR efficiency (PPC: Table 2, H10 to 12). RT^2^ array data was normalized against the house keeping genes by calculating the 2(^-ΔΔCT^) for each gene of interest in the plate. Fold changes of gene expression, heatmap and scatterplot were generated and analyzed by using RT^2^ PCR array data and the analysis web portal (geneglobe. qiagen. com). Groups of genes that had fold changes of more than two in expression when 13 μM EFV treatment samples were compared to control groups were considered significant. The candidate genes were chosen to be validated in an additional RT-qPCR experiment.

**Table 2:** RT^2^ profiler human cell-cycle PCR arrays layout

	1	2	3	4	5	6	7	8	9	10	11	12
**A**	ABL1 NM_005157	ANAPC2 NM_013366	ATM NM_000051	ATR NM_001184	AURKA NM_003600	AURKB NM_004217	BCCIP NM_016567	BCL2 NM_000633	BIRC5 NM_001168	BRCA1 NM_007294	BRCA2 NM_000059	CASP3 NM_004346
**B**	CCNA2 NM_001237	CCNB1 NM_031966	CCNB2 NM_004701	CCNC NM_005190	CCND1 NM_053056	CCND2 NM_001759	CCND3 NM_001760	CCNE1 NM_001238	CCNF NM_001761	CCNG1 NM_004060	CCNG2 NM_004354	CCNH NM_001239
**C**	CCNT1 NM_001240	CDC16 NM_003903	CDC20 NM_001255	CDC25A NM_001789	CDC25C NM_001790	CDC34 NM_004359	CDC6 NM_001254	CDK1 NM_001786	CDK2 NM_001798	CDK4 NM_000075	CDK5R1 NM_003885	CDK5RAP1 NM_016408
**D**	CDK6 NM_001259	CDK7 NM_001799	CDK8 NM_001260	CDKN1A NM_000389	CDKN1B NM_004064	CDKN2A NM_000077	CDKN2B NM_004936	CDKN3 NM_005192	CHEK1 NM_001274	CHEK2 NM_007194	CKS1B NM_001826	CKS2 NM_001827
**E**	CUL1 NM_003592	CUL2 NM_003591	CUL3 NM_003590	E2F1 NM_005225	E2F4 NM_001950	GADD45A NM_001924	GTSE1 NM_016426	HUS1 NM_004507	KNTC1 NM_014708	KPNA2 NM_002266	MAD2L1 NM_002358	MAD2L2 NM_006341
**F**	MCM2 NM_004526	MCM3 NM_002388	MCM4 NM_005914	MCM5 NM_006739	MDM2 NM_002392	MKI67 NM_002417	MNAT1 NM_002431	MRE11A NM_005590	NBN NM_002485	RAD1 NM_002853	RAD17 NM_002873	RAD51 NM_002875
**G**	RAD9A NM_004584	RB1 NM_000321	RBBP8 NM_002894	RBL1 NM_002895	RBL2 NM_005611	SERTAD1 NM_013376	SKP2 NM_005983	STMN1 NM_005563	TFDP1 NM_007111	TFDP2 NM_006286	TP53 NM_000546	WEE1 NM_003390
H^*^	ACTB NM_001101	B2M NM_004048	GAPDH NM_002046	HPRT1 NM_000194	RPLP0 NM_001002	HGDC SA_00105	RTC SA_00104	RTC SA_00104	RTC SA_00104	PPC SA_00103	PPC SA_00103	PPC SA_00103

^*^Gene array controls that only authenticate array results but do not necessarily form part of the genes related to the human cell-cycle pathway.

### Validation of the cell cycle array data

#### Real-time PCR (qPCR)

To validate the expression changes of genes of interest that had fold changes of more than two, the real time PCR was performed. Total RNA from treated and control cells for 24 h and 48 h was extracted using the RNeasy mini kit (Qiagen, USA). The extracted RNA samples were used for reverse transcription in RT-qPCR experiments. Briefly, RNA was reverse transcribed to cDNA using the Maxima First Strand cDNA synthesis kit for RT-qPCR with dsDNase (Thermo Scientific) following the manufacturer’s instructions in an ABI 7500 System. The thermal profile for qPCR was 30s pre-incubation at 95°C for one cycle, followed by 40 cycles of 95°C for 5 s and 60°C for 34 s. Table 3 summarizes the sequences for candidate genes used in this study. The fold changes in expression of targeted genes was normalized using the housekeeping gene GAPDH by the 2^-ΔΔCT^ method [33]. Each experiment was evaluated by three PCR reactions and each experiment was repeated at least three times.

**Table 3:** The sequences of the primers

Gene	Refseq Accession^#^	Direction	Sequence
MAD2L2	NM_006341	Fwd	CGAGTTCCTGGAGGTGGCTGTGCATC
		Rv	CTTGACGCAGTGCAGCGTGTCCTGGATA
CASP3	NM_004346	Fwd	GCTCATACCTGTGGCTGTGTA
		Rv	ATGAGAATGGGGGAAGAGGCA
AURKB	NM_004217	Fwd	AGCAGCGAACAGCCACG
		Rv	GCCGAAGTCAGCAATCTTCA
GAPDH	NM_002046	Fwd	TGCACCACCAACTGCTTAGC
		Rv	GGCATGGACTGTGGTCATGAG

### Ingenuity Pathway Analysis (IPA)

Qiagens’s Ingenuity Pathway Analysis (IPA) has been widely used to model, analyse and understand complex biological systems. In this study, the IPA canonical pathway analysis and core analysis functions were used. The canonical pathway analysis provides insights into data by determining most significantly affected pathways. In this study, IPA canonical pathway analysis was used to reveal significantly affected pathways other than the cell cycle in response to EFV drug treatment. To achieve this, the z core was primarily used to indicate the degree of expression levels, with positive z score denoting upregulation, negative z core representing down-regulation, while zero (0) z core illustrates unchanged gene expression. The core analysis function was used to help build a more complete regulatory picture to better elucidate the biology underlying the studied gene expression profiles.

### Statistical analysis

Fold changes of the transcriptional profiling of the 84 genes expression, scatterplot and heatmap were calculated and generated by using the RT^2^ PCR array data analysis web portal. Although the PCR array was performed once per sample, the arrays were validated by quality check (Table 2) according to the manufacturer’s instructions. Genes of the test group (EFV treated) compared to control group with differences greater than 2-fold (*p* < 0.05) were considered significant, as calculated by the RT^2^ PCR array data analysis web portal. When comparing more than two conditions, data were analyzed by one-way ANOVA, followed by a Tukey’s post hoc test using Graph Pad Prism 5. Values were presented as ± S. E. M for at least three independent experiments. The level of statistical significance was set at *p* < 0.05.

## Abbreviations

DDR: DNA damage response
CCN: cyclin
CDK: cyclin-dependent kinase
CDKN: cyclin-dependent kinase inhibitor
CDKN1/p21: cyclin-dependent kinase inhibitor 1A
MAD2L2: mitotic arrest deficient-like 2
CASP3: apoptosis-related cysteine peptidase
AURKB: Aurora kinase B
TP53: tumor protein p53
ATM: ataxia telangiectasia mutated
GADD45A: growth arrest and DNA-damage-inducible, alpha
